# Obstructive sleep apnea is associated with cognitive impairment in minor ischemic stroke

**DOI:** 10.1007/s11325-022-02575-5

**Published:** 2022-03-19

**Authors:** Ruo-lin Zhu, Chao Ouyang, Ruo-lin Ma, Kai Wang

**Affiliations:** 1 Department of Neurology, The First Affiliated Hospital of Anhui Medical University, No. 218 Jixi Road, 230022 Hefei, People’s Republic of China; 2 Department of Medical Psychology, Anhui Medical University, Hefei, 230022 China; 3 Collaborative Innovation Centre of Neuropsychiatric Disorders and Mental Health, Hefei, 230022 China; 4 Department of Neurology, The Lu’an Civily Hospital, Lu’an 237010, Anhui Province, China

**Keywords:** Stroke, Cognitive impairment, Sleep, Polysomnography

## Abstract

**Objectives:**

Obstructive sleep apnea (OSA) is commonly seen in stroke patients, and its relationship with cognitive impairment remains poorly understood. This study aimed to explore the roles of OSA in cognition impairment in patients with minor ischemic stroke.

**Methods:**

Patients with minor ischemic stroke were consecutively enrolled from January 2020 to May 2021. Every patient underwent polysomnography (PSG) to assess for OSA. Based on the apnea hypopnea index (AHI), patients were grouped into the no OSA (AHI < 5), mild OSA (5 ≤ AHI < 15), and moderate-to-severe OSA (MS OSA, AHI ≥ 15) groups. Neuropsychological assessments were performed to evaluate cognitive function, and the correlations between cognitive function and OSA were investigated.

**Results:**

Of 94 patients, 35 had no OSA, 32 had mild OSA, and 27 had moderate-to-severe OSA. Compared to the no or mild OSA groups, the moderate-to-severe OSA group performed worse on the Chinese version of the Auditory Verbal Learning Test (CAVLT)-Recognition (*p* < 0.001), Digital Span Test (DST)-Backward (*p* < 0.001), Montreal Cognitive Assessment (MoCA) (*p* < 0.001), and Stroop Color and Word Test (SCWT)-Interference (*p* < 0.001). The severity of cognitive impairment was assessed using the MoCA, which was negatively related to the AHI (*p* = 0.041) and lowest SpO2 (*p* = 0.048).

**Conclusions:**

The findings suggest that OSA has significant effects on cognition impairment in patients with minor ischemic stroke and that hypoxemia may be a potential pathophysiological mechanism of OSA-induced cognitive impairment.

## Introduction

Stroke is characterized as a neurology deficit due to clinical, radiological, or pathological evidence of ischemia or hemorrhage with a vascular cause [1]. Epidemiologically, stroke is the 2nd leading cause of mortality and the third leading contributor to serious long-term disability across the globe [2, 3]. About 20–25% of stroke patients are accompanied by serious disability [4]. In China, stroke has imposed a tremendous burden on the health care system. In 2018, the death rate of cerebrovascular diseases was 0.15%, making up 1.57 million deaths across China [5]. Recently, various initiatives have explored innovative stroke prevention strategies to improve the stroke-free survival and quality of life of stroke sufferers.

Cognitive impairment, with a prevalence ranging from 20 to 80%, has been confirmed as a comorbidity of ischemic stroke [6]. Research has shown that cognitive impairment is among the most commonly seen factors that contributes to life disability following stroke, resulting in higher mortality, slower physical restoration, and the loss of economic and social capabilities [7, 8]. Although it is currently widely recognized that cognitive impairment-induced disabilities are frequent after stroke, the nature and magnitude of these impairments and the factors that determine their magnitude are unclear [9]. Previous studies have explored the demographic and psychological variables that influence the onset of cognitive impairment after stroke and reported varied results [10, 11]. However, the mechanism or risk factors of cognitive impairment remain unclear due to heterogeneities in neurocognitive assessment tools, diagnostic criteria for cognitive impairment, and population characteristics, including the cognitive status before stroke onset or the cognitive evaluation intervals after stroke [12]. The identification of novel and potentially reversible risk factors may be useful for clinicians in the prevention and treatment of acute ischemia stroke.

Recently, a growing body of literature has shown an interest in the link between OSA and cognitive impairment. OSA is characterized by repeated nocturnal episodes of partial pharyngeal narrowing (hypopnea) or complete pharyngeal closure (apnea). OSA is an independent risk factor for stroke, and may be a complication of stroke [13, 14]. OSA is often unrecognized in the common population and has high prevalence in stroke sufferers, which is between 30 and 96% depending on the population studied [15–17]. Increasing evidence suggests that OSA may contribute to cognitive impairment in several domains, including concentration, vigilance, episode memory, work memory, and executive functioning [8]. In the population with insomnia and sleep deprivation, OSA has been found to be negatively related to cognitie function [18, 19]. Fortunately, appropriate management, such as continuous positive airway pressure (CPAP) or maxillomandibular advancement devices (MADs), exerts a remarkable effect on multiple cognition domains in OSA sufferers, particularly work memory, long-term verbal memory, and short-term visual spatial memory [21–24].

Minor ischemic stroke is generally defined as a scoring ≤ 5 as per the National Institute of Health Stroke Scale (NIHSS)[20]. Cognitive impairments have been found in more than 80% of patients with minor stroke [26]. To date, limited studies with diverse results have been conducted to evaluate the role of OSA in cognition damage in those patients [21–24]. Most studies recruited acute stroke sufferers with different severities without thorough neural psychological or imaging evaluations [22–24]. Furthermore, various severities of OSA have shown significant differences in prospective memory and sleep efficiency in stroke sufferers [24]. The effects and mechanism of OSA of different severities on cognitive changes in patients with minor stroke remain unclear. Considering the high incidence of OSA and its potential contribution to morbidity and mortality among patients with stroke, we conducted this study to assess the role of OSA in cognition damage in sufferers with minor ischemic stroke.

## Methods

### Study design and participants

The present research’s procedures were approved by the university’s Ethical Board. Every patient offered informed written consent. Patients with ischemia stroke admitted to the Neural Psychiatric Disorders and Mental Health Centre of Anhui Province between January 2020 and May 2021 were invited to participate in our study. The inclusive criteria were as follows: (1) 18 to 75 years of age; (2) ischemic stroke proven via computed tomography (CT) and/or magnetic resonance imaging (MRI) and diagnosed by a neurologist; (3) NIHSS score between 1 and 5 at admission, defined as minor ischemic stroke; and (4) admission between 1 and 7 days after stroke onset.

Patients were excluded if they had (1) serious medical conditions, including respiratory failure and advanced CHF; (2) a history of any cognition impairment or psychiatric comorbidity before the stroke; (3) central sleep apnea, complex sleep apnea, or previously diagnosed OSA; or (4) refusal to participate in all testing. Data on demographic and clinical characteristics, including medical history, medications, and stroke details, were collected. Overnight polysomnography (PSG) was conducted to assess for OSA, and a set of neuropsychological assessments was conducted to evaluate cognitive function.

### Polysomnography

Sleepiness was assessed using the Epworth Sleepiness Scale (ESS) before performing PSG. Each stroke sufferer received PSG using the Alice 6 Diagnosis Platform, Respironics, America for one night at the sleep lab in our department. PSG was conducted over 8 h within 7 days of stroke onset. The parameters investigated included airflow (sensed by both thermistor and pressure transducer), six electroencephalographic leads (F3, F4, C3, C4, O1, and O2), electrooculographic, electrocardiographic, electromyographic (chin and leg muscles) measurements, thoracoabdominal respiratory effort, snoring, body position, blood oxygen saturation, and pulse rate.

Apnea was defined as air flow cessation ≥ 90% lasting for ≥ 10 s. Apnea with ongoing chest and abdomen effort, no chest and abdomen movements, and with initial lack of motion followed by respiratory effort were categorized as obstructive, central, and mixed apneas. Hypopnea was defined as a reduction in air flow or a reduction of thoracic and abdominal movement amplitude of 50% for ≥ 10 s with an oxygen desaturation ≥ 3%. The apnea hypopnea index (AHI) was the average of apneas and hypopneas per hour of sleep. The clinical diagnosis of OSA was made in patients with an AHI ≥ 5 by PSG [25]. The sufferers were grouped into the no OSA group (AHI < 5), which served as the control group in this research, mild OSA group (AHI 5–15), and moderate-to-severe OSA (MS OSA) group (AHI ≥ 15).

The PSG parameters of sleep structure variables (arousal index, sleep efficiency, total sleep time (TST), proportion (%) of sleep duration in stage 3 sleep, and proportion (%) of sleep duration in the rapid eye movement (REM) stage) and hypoxia and disordered breathing (proportion (%) of nighttime spent with an oxyhemoglobin saturation < 90% (TSat90), lowest SpO2, average SpO2, and oxygen desaturation index (ODI)) of all patients were collected.

### Cognition assessment

Every patient ompleted a set of standardized neuropsychology assessments to evaluate global cognition and clinical symptoms within 7 days after stroke. All tests were carried out in a quiet and separate room outside the stroke unit. These assessments included the following:

1. the CAVLT was utilized to assess functions of memory, including instantaneous memory, delayed memory, and recognition memory functions;
2. the Digital Span Test (DST), which includes forward and backward tests, used as a tool for investigating verbal attention and working memory;
3. the Montreal Cognitive Assessment (MoCA) was utilized to assess multiple cognition domains, such as short-term memory, attention and work memory, visual spatial capability, executive function, concentration, linguistic skill, and spatiotemporal orientation;
4. the Hamilton Anxiety Rating Scale (HAMA) was used to evaluate the severity of depressive symptoms;
5. the Hamilton Rating Scale for Depression (HAMD) was used to evaluate anxiety; and
6. the Stroop Color Word Test (SCWT), which comprises the Stroop color, word, and interference tests, was employed to evaluate executive function.

### Statistical analysis

All data were analyzed via SPSS 23.0 (America). Standard descriptive statistics were used to summarize the clinical characteristics and sleep and cognitive assessments of all participants. The differences in the clinical characteristics, polysomnography parameters, and cognitive assessment among the 3 groups were analyzed via ANOVA. The post hoc (LSD) tests for multiple comparisons were also performed to compare the difference of polysomnography parameters and cognitive functions among the 3 groups. A binary logistic regression analysis with the “enter” approach was utilized to forecast OSA on the foundation of the AHI and potential predictors. Furthermore, the patients were classified into two groups that exhibit cognition damage (MoCA < 26) and normal cognition function (MoCA ≥ 26). The polysomnography predictor of cognitive impairment based on MoCA in stroke patients was also evaluated by a logistic regression analysis with adjustments for confounding covariates. *p* < 0.05 was used to signify statistical significance.

## Results

### Clinical features

**Fig. 1:**
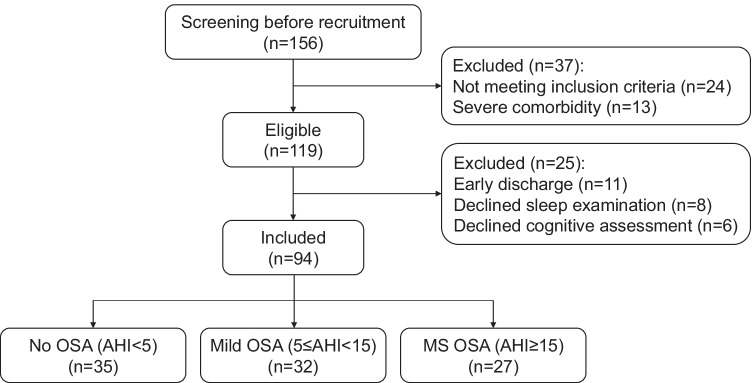
Inclusion and exclusion criteria of selected patients

To determine the potential correlation between OSA and cognition damage in ischemia stroke sufferers, 156 healthy adults were initially recruited from Anhui Medical University, and 94 stroke sufferers were selected (Fig. 1). Each sufferer was diagnosed with ischemia stroke for the first time. Thirty-five stroke patients without OSA (No OSA) were taken as controls, accounting for 37% of this population; 32 sufferers (34%) were diagnosed with mild OSA, and 27 (29%) sufferers were diagnosed with medium-to-severe OSA (MS OSA). Their demography and clinic features are summarized in Table 1.

**Table 1:** Baseline characteristics in patients with minor ischemic stroke

	No OSA (*n* = 35)	Mild OSA (*n* = 32)	MS OSA (*n* = 27)	*p* value
Age, years, mean (SD)	57.6 (10.3)	55.9 (6.8)	55.8 (8.0)	0.653
Male, *n* (%)	26 (74%)	22 (69%)	18 (67%)	0.795
Education, years, mean (SD)	9.5 (2.6)	8.8 (2.7)	7.7 (3.1)	0.146
Hypertension, *n* (%)	15 (43%)	18 (56%)	21 (78%)	0.021
Atrial fibrillation, *n* (%)	11 (31%)	9 (28%)	9 (33%)	0.910
Diabetes, *n* (%)	8 (23%)	11 (34%)	12 (44%)	0.201
Myocardial infarction, *n* (%)	10 (29%)	14 (44%)	7 (26%)	0.280
Smoking status, *n* (%)	10 (29%)	19 (59%)	15 (56%)	0.022
Drinking status, *n* (%)	10 (29%)	9 (28%)	7 (26%)	0.972
Antiplatelets, *n* (%)	8 (23%)	10 (32%)	8 (30%)	0.725
Anticoagulants, *n* (%)	5 (14%)	7 (22%)	7 (26%)	0.514
Statin, *n* (%)	7 (20%)	8 (25%)	7 (26%)	0.837
NIHSS, mean (SD)	2.8 (1.6)	2.7 (1.5)	2.4 (1.5)	0.597
Stroke in cortex, *n* (%)	9 (26%)	11 (34%)	11 (41%)	0.458
Stroke in cerebellum, *n* (%)	16 (46%)	16 (50%)	11 (41%)	0.782
Stroke in brain stem, *n* (%)	10 (29%)	5 (16%)	5 (19%)	0.406
HAMA	4.6 (1.8)	5.1 (1.7)	4.0 (1.1)	0.132
HAMD	5.1 (1.9)	5.0 (1.7)	4.3 (1.1)	0.103

*OSA*, obstructive sleep apnea; *MS OSA*, moderate-to-severe OSA; *NIHSS*, National Institutes of Health Stroke Scale; *HAMA*, Hamilton Anxiety Rating Scale; *HAMD*, Hamilton Rating Scale for Depression

We continued to explore which clinical parameters independently contributed and how strong their contribution was to the onset of OSA in ischemic stroke patients. All sufferers in the presence and absence of OSA measured by the AHI were pooled, and binary logistic regression analyses were performed to test whether or not these baseline characteristics (including demographics, medical history, chronic medications, and stroke location) independently predicted the onset and severity of OSA. Our analysis showed that the risk factors for the onset of OSA included sex, hypertension, and smoking (odds ratio [OR] = 0.17, 95% CI 0.04 to 0.74, *p* = 0.02; OR = 4.61, 95% CI 1.45 to 14.69, *p* = 0.01; and OR = 9.18, 95% CI 2.32 to 36.28, *p* = 0.00, respectively). However, these parameters were not remarkably related to the different severities of OSA (Table 2).

**Table 2:** Possible predictors of risk of OSA based on AHI in patients with minor ischemic stroke

	No OSA vs. OSA			Mild OSA vs. MS OSA		
	*β*	OR (95% CI)	*p* value	*β*	OR (95% CI)	*p* value
Age	− 0.02	0.98 (0.92–1.05)	0.60	− 0.04	0.97 (0.88–1.06)	0.46
Gender	− 1.77	0.17 (0.04–0.74)	0.02	− 0.16	0.85 (0.09–7.67)	0.89
Education	− 0.12	0.88 (0.73–1.08)	0.22	− 0.16	0.85 (0.68–1.07)	0.17
Hypertension	1.53	4.61 (1.45–14.69)	0.01	1.18	3.25 (0.87–12.14)	0.08
Atrial fibrillation	− 0.11	0.90 (0.29–2.78)	0.85	0.17	1.18 (0.33–4.27)	0.80
Diabetes	0.80	2.23 (0.68–7.33)	0.19	0.19	1.21 (0.35–4.22)	0.76
MI	0.42	1.52 (0.46–5.06)	0.49	− 0.75	0.47 (0.14–1.6)	0.23
Smoking	2.22	9.18 (2.32–36.28)	0.00	− 0.15	0.86 (0.11–6.79)	0.89
Drinking	− 0.51	0.60 (0.17–2.12)	0.43	− 0.55	0.57 (0.13–2.56)	0.47
Antiplatelets	0.22	1.25 (0.36–4.35)	0.73	− 0.44	0.64 (0.17–2.44)	0.52
Anticoagulants	0.53	1.70 (0.38–7.49)	0.49	0.35	1.42 (0.33–6.15)	0.64
Statin	0.52	1.68 (0.46–6.1)	0.43	0.11	1.11 (0.26–4.8)	0.89
Stroke location	− 0.55	0.58 (0.27–1.22)	0.15	− 0.27	0.76 (0.29–2)	0.58

The associations of clinical variable with the onset of OSA were evaluated by logistic regression. *OSA*, obstructive sleep apnea; *MS OSA*, moderate-to-severe OSA; *β*, partial regression coefficient; *OR*, odds ratio; *95% CI*, 95% confidence interval

### Sleep studies

Before PSG, all patients completed the ESS questionnaire to evaluate their daytime sleepiness. The results revealed no remarkable differences among the stroke sufferers in the 3 groups (*p* = 0.553). Among those with OSA, especially medium-to-severe OSA, there were abnormal sleep parameters, including a higher arousal index, AHI, and worse sleep efficiency, compared to the those with no OSA (*p* < 0.01). Furthermore, the OSA sufferers had higher rates of hypoxia and disordered breathing as manifested by lower SpO2 and higher incidences of ODI and TSat90 (*p* < 0.001). However, different sleep architecture parameters were not found between the ischemic stroke sufferers in the presence and absence of OSA (Table 3).

**Table 3:** Polysomnography parameters of enrolled stroke patients

Variable	No OSA (*n* = 35)	Mild OSA (*n* = 32)	MS OSA (*n* = 27)	*p* value
ESS	7.5 (4.4)	6.7 (3.5)	6.5 (2.9)	0.553
Arousal index, events/h	21.3 (4.5)	22.9 (6)	32.7 (9.5)^†,‡^	< 0.001
AHI, events/h	2.45 (0.55)	9.62 (1.69)*	19.29 (1.69)^†,‡^	< 0.001
Lowest SpO2, %	80.9 (2.4)	76.8 (2.7)*	75.9 (3.2)^†^	< 0.001
Mean SpO2, %	91.4 (4.0)	87.3 (3.1)*	82.4 (4.6)^†,‡^	< 0.001
ODI (≥ 3%), events/h	4.3 (1.3)	8.4 (1.8)*	15.2 (4.8)^†,‡^	< 0.001
Sleep architecture, % TST				
Stage N1	13.5 (3.9)	14.0 (2.4)	14.8 (2.1)	0.263
Stage N2	52.3 (9.0)	52.9 (6.4)	51.9 (4.6)	0.857
Stage N3	20.8 (3.2)	20.6 (2.0)	19.2 (3.6)	0.112
Stage REM	10.8 (3.1)	11.2 (3.3)	10.3 (2.5)	0.507
Sleep efficiency, %	69.6 (6.4)	65.4 (7.6)*	64.1 (6.7)^†^	0.006
TST, min	295 (53)	304 (64)	302 (56)	0.807
TSat90	4.9 (2.0)	7.3 (1.6)*	14.9 (2.5)^†,‡^	< 0.001

Data are expressed as mean with standard deviation. *Significant difference between No OSA with Mild OSA; ^†^Significant difference between No OSA with MS OSA; ^‡^Significant difference between Mild OSA with MS OSA

*OSA*, obstructive sleep apnea; *MS OSA*, moderate-to-severe OSA; *ESS*, Epworth Sleepiness Scale; *AHI*, apnoea hypopnoea index; *ODI*, oxygen desaturation index with oximetry recording; *REM*, rapid eye movement; *SpO2*, pulse oxygen saturation; *TST*, total sleep time; *TSat90*, percent of night-time spent with an oxygen saturation of < 90%

### Cognitive functions and obstructive sleep apnea

Table 4 describes the performance of patients with different severities of OSA on measures of cognitive functions. The performance of the sufferers with mild and medium-to-serious OSA on the CAVLT-Recognition, DST-Backward, and SCWT-Interference tests was lower in contrast to the sufferers with no OSA (*p* < 0.001). The MoCA is known to be more comprehensive and precise than other cognitive tests, and the scores decreased progressively as the OSA severity increased (*p* < 0.001 for trend). If MoCA score < 26 is indicative of cognitive impairment, the binary logistic regression analysis adjusted for the PSG parameters revealed a remarkable correlation between loss of cognition and AHI scores as well as lower SpO2 values (*p* < 0.05) (Table 5).

**Table 4:** Cognitive function in patients with minor ischemic stroke

	No OSA (*n* = 35)	Mild OSA (*n* = 32)	MS OSA (*n* = 27)	*p* value
CAVLT-Immediate	10.6 (1.4)	10.0 (1.2)	10.0 (1.5)	0.097
CAVLT-Delay	9.7 (1.5)	9.1 (1.2)	9.0 (1.6)	0.125
CAVLT-Recognition	8.8 (1.1)	8.2 (1.4)*	6.9 (1.0)^†,‡^	< 0.001
DST-Forward	5.1 (1.4)	5.4 (0.9)	5.3 (1.0)	0.469
DST-Backward	4.4 (1.1)	5.2 (0.8)*	3.9 (0.7)^‡^	< 0.001
MoCA	26.5 (2.8)	24.3 (2.1)*	21.9 (2.4)^†,‡^	< 0.001
SCWT-Color test	25.2 (3.0)	25.9 (1.0)	26.4 (3.0)	0.143
SCWT-Word test	30.0 (4.1)	29.7 (2.3)	31.4 (1.7)	0.064
SCWT-Interference test	35.9 (1.9)	42.1 (1.7)*	58.2 (1.3)^†,‡^	< 0.001

Data are expressed as mean with standard deviation. *Significant difference between No OSA with Mild OSA; ^†^Significant difference between No OSA with MS OSA; ^‡^Significant difference between Mild OSA with MS OSA

*OSA*, obstructive sleep apnea; *MS OSA*, moderate-to-severe OSA; *CAVLT*, Chinese version of the Auditory Verbal Learning Test; *DST*, Digital Span Test; *MoCA*, Montreal Cognitive Assessment; *SCWT*, Stroop Color Word Test

**Table 5:** Cognitive impairment based on MoCA in stroke patients according to polysomnography results

	*β*	OR (95% CI)	*p* value
Arousal index	− 0.023	0.98 (0.858–1.113)	0.729
AHI	− 0.043	1.42 (1.056–1.706)	0.041
Lowest SpO2	0.311	1.37 (1.003–1.857)	0.048
Mean SpO2	0.004	1.00 (0.823–1.226)	0.966
ODI	− 0.134	0.87 (0.668–1.144)	0.327
Stage N1	0.35	0.96 (0.687–1.337)	0.081
Stage N2	− 0.037	0.96 (0.874–1.062)	0.456
Stage N3	0.079	1.08 (0.788–1.487)	0.625
Stage REM	− 0.011	0.99 (0.79–1.239)	0.927
Sleep efficiency	− 0.006	0.99 (0.896–1.102)	0.906
TST	0.002	1.00 (0.986–1.018)	0.819
TSat90	− 0.231	0.79 (0.517–1.22)	0.292
ESS	0.097	1.10 (0.886–1.371)	0.383

*AHI*, apnoea hypopnoea index; *ODI*, oxygen desaturation index with oximetry recording; *ESS*, Epworth Sleepiness Scale; *REM*, rapid eye movement; *SpO2*, pulse oxygen saturation; *TST*, total sleep time; *TSat90*, percent of night-time spent with an oxygen saturation of < 90%; *β*, partial regression coefficient; *OR*, odds ratio; *95% CI*, 95% confidence interval

## Discussion

OSA is a commonly seen comorbidity in stroke sufferers that reportedly exaggerates cognition damage [31]. In this study, there was a high prevalence of stroke survivors suffering from OSA (63% (59/94)), which is consistent with the outcomes of past research [28, 32, 33]. Hypertension and smoking were ommon in the mild and medium-to-serious OSA groups more frequently compared to the no OSA group. This finding was expected as both factors are known risk factors for OSA [34]. Compared with the patients without OSA, the OSA sufferers demonstrated a greater prevalence of hypoxia and disordered breathing, including arousal index, SpO2, ODI, sleep efficiency, and TSat90 (*p* < 0.001). Lower cognitive performance (including the CAVLT, DST, MoCA, and SCWT) was also identified in the sufferers with mild and medium-to-serious OSA in contrast to the sufferers with no OSA. The regression analysis revealed a link among MoCA, lower AHI values, and the lowest SpO2 (*p* < 0.05). Cognitive impairment was not associated with the other clinical characteristics, such as stroke type or classification, which is consistent with previous studies [27].

A primary finding of this study is that OSA is linked to a poorer neurological status, including memory and executive function, at the time of admission in patients with ischemia stroke. Those discoveries are backed by past research that showed that OSA was related to an impaired functional status at discharge and 3 and 12 months following stroke [26–29]. However, some results did not completely coincide with past research. Zhang et al. [29] also showed that time- and event-based prospective memory was damaged independently by OSA in stroke sufferers. Slonkova et al. [28] demonstrated poorer memory in the OSA group in contrast to the no OSA group of stroke patients. Aaronson et al. [27] discovered that OSA sufferers following stroke presented inferior results in examinations of concentration, executive function, visual perception, psychomotor capability, and intelligence in contrast to the sufferers with no OSA, while no differences were seen in memory between the sufferers in the 2 groups. These differences might be explained by the neuropsychology assessments conducted at different times (4 weeks vs. 3 and 12 months after admission), and different neuropsychologic tools. On the contrary, Kaneko et al. [35] did not see differences in the neurological status (measured with the Canadian Neurological Scale) between stroke sufferers in the presence and absence of OSA upon admission to a stroke rehabilitation unit, even though a slightly lower score was found in the patients with OSA. The small size of the no OSA group might have accounted for these results.

To date, most research exploring the association between sleep disorders and decreased cognition have focused on sleep apnea after stroke, and studies investigating the association between sleep duration and architecture and decreased cognition after stroke are insufficient [36]. Our team explored the potential pathophysiological mechanisms mediating OSA-associated cognitive impairment by analyzing the sleep time and structure in stroke patients. Our results revealed a higher occurrence of sleep disorders, including both airflow of respiratory tracts (arousal index, AHI, and ODI) and blood oxygen levels (SpO2 and TSat90), and lower sleep efficiency in the patients with mild and medium-to-severe OSA compared with the patients with no OSA in our experiments [37, 38]. The logistic regressive analyses further revealed that the AHI and lowest SpO2 were the most important risk factors for cognition damage based on the MoCA.

Certain limitations of our research deserve disclosure. First, the cross-sectional research design prevented causal inference between cognitive impairment and OSA in patients with stroke. Prospective cohort study is necessary in future investigations. Second, the sample size of our study was relatively small, which affects the conclusions. More research based on a bigger sample size are warranted. Third, there were deviations in the prevalence of hypertension and smoking status in the stroke sufferers in the presence and absence of OSA, and these differences may have affected the comparison of the polysomnography and neuropsychology assessments among the groups.

## Conclusions

Findings of this study suggest that OSA contributes to the cognitive impairment in patients with minor ischemic stroke. The data further suggest that hypoxemia may underline pathological and OSA-triggered cognition impairment. These discoveries highlight the significance of OSA as a possible predictive factor for cognitive impairment.
